# The Use of Antisense-Mediated Inhibition to Delineate The Role of Inflammatory Agents in The Pathophysiology of Spinal Cord Injury

**DOI:** 10.1100/tsw.2002.63

**Published:** 2002-05-29

**Authors:** Damien D. Pearse, Francisco C. Pereira, Katina Chatzipanteli, Francisco W. Dalton Dietrich, Mary Bartlett Bunge

**Affiliations:** The Miami Project to Cure Paralysis, Department of Cell Biology and Anatomy, University of Miami School of Medicine, Miami, FL, USA; The Miami Project to Cure Paralysis, Department of Neurological Surgery, USA; Department of Anatomy, University of Sao Paulo, Brazil

## Abstract

Injuries to the central nervous system (CNS) usually lead to a potent and acute inflammatory response[1]. During this period, glia and immune cells respond to chemical cues associated with the debris of lysed neurons, disrupted axons, and a broken blood-brain-barrier by releasing a battery of cytokines including tumor necrosis factor-*α* (TNF-*α*) and, interleukin-β (IL-1β) as well as reactive oxygen species such as nitric oxide (NO^-^)[2]. The secretion of these factors may be primarily responsible for secondary damage to surrounding uninjured tissue that potentiates the initial injury[3]. Antisense oligonucleotides (ASOs) are designed to hybridize to specific regions of specific mRNAs. Hybridization of the oligonucleotide to the mRNA then interferes with the normal processing of that mRNA at the ribosome or targets the RNA duplex for cleavage by the RNA digestive enzyme, ribonuclease H, resulting in greatly reduced expression of the coded protein. This effectively reduces the amount of corresponding translated protein product and experiments can be designed to examine the requirement of particular inflammatory agents in eliciting specific deleterious responses after injury, e.g., cell death.

**METHODS.**

**Transfection Efficiency**. Tagged (5’ fluorescein) ASOs were made from 18-21 base sequences of the different cytokine cDNAs (TNF-α or IL-1β) or the inducible nitric oxide synthase cDNA and mixed base oligonucleotides of the same nucleotide composition for controls. These constructs were used to examine specific cellular uptake, compartmentalization, and transfection efficiency either alone or in the presence of transfecting agents, LipofectAMINE or NOVAfector. These were either directly injected or superfused onto the dorsal spinal cord after opening the dura mater. Administration occurred 15 min after moderate contusion of thoracic spinal cord (T10) of adult Fischer rats. Animals were perfused 6, 24, or 72 h later and fluorescein labeling was compared to cellular markers (double-labeling), GFAP (astrocytes), NeuN (neurons), ED1 (activated macrophages and microglia), MPO (neutrophils) and APC+/GFAP- (oligodendrocytes) staining with rhodamine $2^{o}$Ab in transverse tissue sections spanning the lesion epicenter and caudal and rostral to this.

**Protein Inhibition**. The most efficacious method of transfection was then used with unlabeled oligonucleotides to examine the efficiency of inhibition. Following injury and administration (6, 24, or 72 h), spinal cord blocks of 2-mm lengths were removed from the epicenter and caudal and rostral regions and assayed for protein levels by ELISA against total protein content or by cellular expression by immunochemistry.

**Cell Death**. Sections were also examined immunohistologically for evidence of early cell death (phosphatidylserine externalization or plasmalemmal breakdown) using the annexin apoptosis-necrosis system and a combination of specific cell markers.

**RESULTS.** Transfection efficiency was significantly greater by spinal cord superfusion than by single intraspinal cord injection. The transfecting agents did not enhance ASO transfection rate and appeared to be toxic to several cell types. FITC-ASO uptake was primarily localized to astrocytes, oligodendrocytes, neutrophils, and dorsal horn neurons. Significant reductions in specific protein expression were observed with the ASO constructs for up to 72 h at the injury site. TNF-α ASO-mediated inhibition of TNF-α (reduced by $86.7 \pm 8.1\%$, $p < 0.01$) resulted in a profound loss (>70%) of GFAP+ and S100-β+ astrocytes within 6 h and for up to 24 h (almost exclusively by necrosis), both at the injury epicenter and up to 5 mm rostrally and caudally. The remaining GFAP+ astrocytes exhibited deformed cell processes and an enlarged cell body: they were found almost exclusively in ventral white matter. The profound loss of astrocytes after TNF-α inhibition led to a decreased architectural integrity of the spinal cord. With reduced astrogliosis, many more NeuN+ neurons were observed in the dorsal horn (68.4%), intermediate layers (48.3%), and the ventral horn (23.7%) compared to controls. Oligodendrocyte numbers were also greater in the injury site and in adjacent areas after TNF-α ASO administration. This would suggest that the secretion of TNF-α is trophic for astrocytes after injury, increasing their reactivity in a survival response. In SCI, the secretion of TNF-α may allow astrocyte survival to take precedence over neuronal and oligodendrocyte survival and could be important for ensuring that the structural integrity of the spinal cord after injury is better preserved. Inhibition of $NO^-$ formation through the antisense knockdown of iNOS (>90%) slightly reduced the number of neutrophils present at the injury site 24 h after injury. The degree of tissue damage, edema and BBB disruption was significantly less and there was a dramatic reduction within the injury site of accumulated erythrocytes after iNOS ASO.

**SIGNIFICANCE OF FINDINGS.** Antisense inhibition of specific inflammatory agents after SCI may allow the deduction of the roles of these factors in the progression of the injury. The current study has addressed only three of a multitude of inflammatory agents and other proteins that are expressed following CNS injury and whose functions in injury processes are poorly understood. Antisense inhibition could also offer a specific targeting therapy to reduce inflammatory-mediated damage acutely after SCI. Indeed, many clinical trials are presently underway for the use of antisense targeting as a therapeutic strategy for a range of diseases from cytomegalovirus to cancer.

**ACKNOWLEDGEMENTS.** We thank Raisa Puzis, Paulo Diaz, Santiago Castro, and Alexander Marcillo for outstanding assistance. Funding from the Christopher Reeve Paralysis Foundation and The Miami Project.
